# Effects of Prenatal Ursodeoxycholic Acid and Probiotic Supplementation on Stress Parameters, Immune Response and Lamb Survival Rates in Ewes

**DOI:** 10.1002/vms3.70859

**Published:** 2026-07-29

**Authors:** M. Burak Ates, Fatih Aladag, Durmus Hatipoglu

**Affiliations:** ^1^ Faculty of Veterinary Medicine, Department of Pathology Selcuk University Konya Türkiye; ^2^ Bahri Dagdas International Agricultural Research Institute Konya Türkiye; ^3^ Faculty of Veterinary Medicine, Department of Physiology Selcuk University Konya Türkiye

**Keywords:** colostrum, ewes, immunoglobulin G (IgG), lambs, probiotics

## Abstract

To evaluate the effects of prenatal ursodeoxycholic acid (UDCA) and probiotics—alone or combined—on colostrum quality and lamb survival in ewes. Forty Middle Anatolian Merino ewes were randomized to control, UDCA (5 mg/kg/day), PRO (probiotic 0.2 mL/kg; 6.2 × 10^7^ colony‐forming unit (CFU)/mL ≈ 1.24 × 10^7^ CFU/kg) or UDCA + PRO for the last month of gestation. Maternal blood/colostrum was sampled pre‐ and postpartum; lamb serum was sampled at 24 h to assess immunoglobulin G (IgG), total antioxidant capacity (TAC), total oxidant status (TOS) and colostrum composition (IgG, fat, protein and lactose). Growth and survival were monitored to weaning. Maternal treatments significantly affected colostral IgG (*p* = 0.017; highest in UDCA + PRO). In lambs, IgG (*p* = 0.037), total protein (TP) (*p* = 0.021) and albumin (*p* = 0.011) differed by group, favouring UDCA + PRO. Survival improved with treatment (*p* = 0.0014). Across groups, time effects were observed for triglycerides (*p* < 0.001), cholesterol (*p* = 0.002), gamma‐glutamyl transferase (GGT) (*p* < 0.001) and bilirubin (*p* = 0.004). Treatment‐specific changes included PRO increasing colostrum fat (*p* = 0.010) and lactose (*p* = 0.050) and lowering maternal GGT (*p* = 0.021) while elevating TOS (*p* = 0.032) and TAC (*p* = 0.039); UDCA + PRO increased postpartum TOS (*p* = 0.034) and TAC (*p* = 0.005); UDCA altered glucose and triglycerides (both *p* = 0.015). In hierarchical regression, maternal IgG and colostrum IgG positively predicted neonatal IgG (*p* = 0.005). A higher order interaction suggested attenuation of transfer efficiency when maternal IgG, maternal TP and colostrum IgG were simultaneously high (*p* = 0.088). Combined UDCA + PRO most consistently enhanced passive immunity indicators and survival.

## Graphical abstract

Prenatal ursodeoxycholic acid (UDCA) and probiotic supplementation, administered alone or combined, modulate colostrum quality and neonatal immunity in ewes. Combined UDCA + PRO most consistently increases colostral IgG, enhances neonatal IgG levels, and improves lamb survival. Maternal and colostral IgG positively predict neonatal passive immunity, highlighting the synergistic effect of combined supplementation during late gestation.

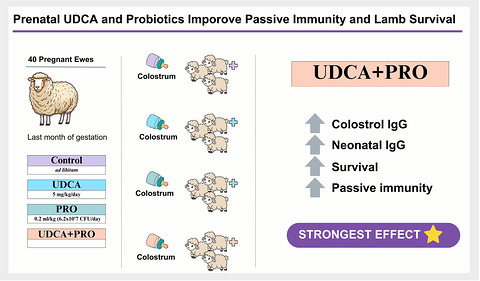

**Abbreviations**
CFU: colony‐forming unit
IgG: immunoglobulin G
IL: interleukin
TAC: total antioxidant capacity
TLR: toll‐like receptor
TNF‐α: tumour necrosis factor‐alpha
TOS: total oxidant status
UDCA: ursodeoxycholic acid

## 1 Introduction

In ruminants, the complex structure of the syndesmochorial placenta limits the transfer of immunoglobulins, mainly immunoglobulin G (IgG), from the mother to the foetus, resulting in neonates being born (Godden 2008; Zhu et al. 2021). Therefore, newborn ruminants must receive colostrum within the first few hours postpartum to acquire passive immunity through the uptake of IgG by enterocytes (Yang et al. 2019). The absorption of colostral IgG in newborn ruminants is most efficient within the first 6 h, decreases between 6 and 12 h and ceases around 24 h after birth (Cabral et al. 2012). Nutritional supplementation of ewes during pregnancy can influence the quantity and quality of colostrum. This impact is linked to the development of the mammary gland, the secretion of hormones that regulate lactogenesis and parturition and the energy intake of the ewe in meeting the metabolic demands of pregnancy, thereby affecting colostrum availability for the lamb (Banchero et al. 2015). Moreover, maternal nutrition during late gestation has been shown to exert long‐term effects on offspring physiology and adaptability, emphasizing the broader implications of prenatal dietary interventions (Laporte‐Broux et al. 2011).

Probiotics are feed additives composed of non‐pathogenic, gram‐positive and anaerobic bacteria that modulate gut microbiota and suppress pathogenic organisms (Alp and Kahraman 1996; Arda et al. 1997). They enhance both humoral and cell‐mediated immunity, thus improving resistance to infections. In ruminants, dietary probiotic supplementation has been linked to improved immune status and productivity (Saleem et al. 2025). For example, *Bacillus subtilis* administration in Barki lambs increased serum lysozyme activity (Mousa et al. 2019), whereas a probiotic blend elevated total protein (TP) and immunoglobulin levels in Holstein calves (Wang et al. 2022). Similar improvements in IgG concentrations were observed in lambs receiving *B. subtilis*, *Bacillus licheniformis* and *Lactobacillus plantarum* (Chen et al. 2021), and significant increases in IgA, IgM and IgG were reported with the addition of *B. licheniformis* and *Saccharomyces cerevisiae* (Jia et al. 2018). In addition to their immunostimulatory role, probiotics promote mucosal integrity by stimulating mucin production and tight junction expression, thereby reducing intestinal permeability (Saleem et al. 2024). Although the immunomodulatory effects of probiotics in both ewes and lambs are well established, the impact of maternal probiotic supplementation during gestation on neonatal immune development remains largely unclear. Although bacteria have been detected in the meconium of newborn calves (Alipour et al. 2018), suggesting possible prenatal microbial exposure via maternal transfer (Funkhouser and Bordenstein 2013), this idea has been further supported by Elolimy et al. (2020), who identified hindgut bacterial populations in neonatal calves immediately after birth, implying a possible dam‐to‐foetus efflux of microbes. In contrast, Malmuthuge and Griebel (2018) reported that the foetal intestine and intrauterine environment remain sterile during the third trimester of gestation, and that gastrointestinal colonization begins only at parturition following rupture of the amniotic membrane. Taken together, these conflicting findings highlight that current evidence for in utero microbial colonization remains inconclusive and largely speculative. The extent to which maternal probiotic supplementation during pregnancy can affect the immunological development of offspring thus remains uncertain and warrants further investigation.

Bile acids are essential microbial metabolites that modulate the homeostasis of the intestinal immune microflora (Song et al. 2020). In humans, ursodeoxycholic acid (UDCA) is a secondary bile acid derived from the metabolism of the primary bile acid, chenodeoxycholic acid (Keely et al. 2019). UDCA has been approved as a standard treatment for primary biliary cholangitis (Carey et al. 2015). Additionally, it has been shown that UDCA treatment improves dysplasia in patients with inflammatory bowel disease and significantly reduces the risk of advanced adenoma through modulation of the gut microbiome (Pearson et al. 2019). Experimental studies have demonstrated that UDCA significantly ameliorates colitis induced by dextran sodium sulphate in mice, characterized by decreased mucosal erosion, crypt destruction and inflammatory cell infiltration in histological examination (Deng et al. 2024; Martínez‐Moya et al. 2013; Zhang et al. 2025). Furthermore, pro‐inflammatory cytokine mRNA expression of interleukin‐1β (IL‐1β), IL‐6 and tumour necrosis factor‐alpha (TNF‐α) in the terminal ileum was significantly reduced in UDCA‐treated mice. It has also been shown that in addition to its hepatoprotective effect, UDCA reshapes intestinal homeostasis and prevents colitis and colitis‐associated colorectal cancer by promoting Akkermansia and Lactobacillus colonization, which forms a mucus layer along the intestinal wall (He et al. 2023). Moreover, in experimental rat models of ethanol‐induced liver injury, UDCA treatment has been reported to improve liver morphology, reduce serum marker enzyme activities and hepatic triglyceride content and normalize oxidative stress markers (Lukivskaya et al. 2006). Thus, due to its potential to reduce oxidative stress, modulate the microbiota and enhance nutrient utilization, UDCA is hypothesized to improve colostrum quality and facilitate the absorption of immunoglobulins in lambs during the prenatal transition period.

This study aims to comprehensively investigate the effects of UDCA, probiotics and their combinations during the prenatal period in ewes exposed to the inherent physiological challenges of pregnancy without introducing additional stress factors. The primary objective is to determine how these treatments influence colostrum composition, enhance serum IgG concentrations and improve immune responses in ewes and their offspring. By strengthening the neonatal immune system from birth to weaning, the interventions are hypothesized to provide significant benefits in terms of health and survivability. By mitigating these risks through enhanced maternal immunity and optimized colostrum quality, the treatments are expected to improve lamb survival rates. This research is driven by the need to develop effective prenatal strategies that not only support the health of the ewe but also contribute to the sustainability of sheep farming by ensuring the survival and health of the next generation.

## 2 Materials and Methods

### 2.1 Ethic Statement

This study was conducted under standard commercial sheep farming conditions at the Bahri Dağdaş International Agricultural Research Institute farm (Konya, Türkiye). The experimental protocol was approved by the Local Ethics Committee for Animal Experiments of the Bahri Dağdaş International Agricultural Research Institute (Approval No.: 161, Approval Date: 29.09.2023).

### 2.2 Animal Material and Experimental Design

**FIGURE 1:**
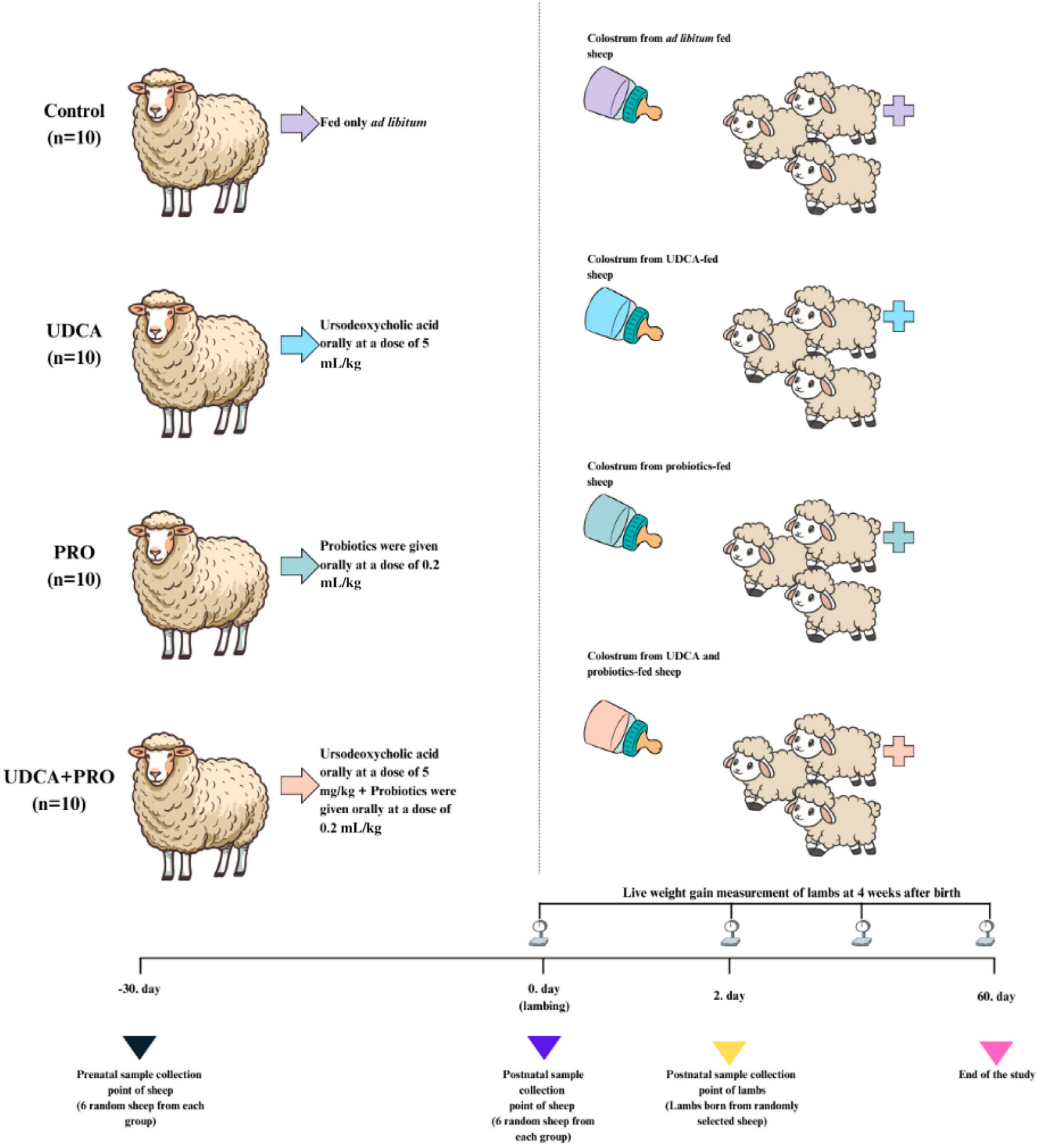
Experimental design illustrating the effects of prenatal ursodeoxycholic acid and probiotic supplementation.

The experimental material consisted of 40 Middle Anatolian Merino ewes in their second parity, housed at the Bahri Dağdaş International Agricultural Research Institute. All ewes were kept in semi‐open barns and fed a standard ration. Following breeding using artificial insemination, pregnancy was confirmed, and 40 pregnant ewes with similar or close conception dates were transferred to separate pens 1 month before parturition. During late gestation, ewes were predominantly housed indoors with restricted access to grazing. Considering the physiological demands of this period and the additional requirements associated with twin pregnancies, the nutrient needs were determined on the basis of National Research Council guidelines. Individual dry matter intake (DMI) was calculated on the basis of the known dry matter (DM) content and the daily ration allocation for each feed component. Each ewe received a total mixed ration (TMR) comprising 850 g/day of sheep dairy concentrate feed (88.5% DM), 500 g/day of corn silage (25% DM), 500 g/day of wheat straw (85% DM), 350 g/day of alfalfa hay (90% DM) and 100 g/day of corn grain (88% DM). On the basis of these values, the daily DMI per ewe was calculated as 1.705 kg/day. No feed refusals were observed during the trial period, and all rations were completely consumed. Although individual intake was not directly measured, the equal feed allocation and complete consumption across animals provide a reasonable and valid basis to assume consistent nutrient intake among groups (NRC 2007). The dietary nutrient composition for late‐gestation ewes and the specific composition of the sheep dairy concentrate feed are detailed in Table 1. In light of the increased water requirements during this period, clean water was provided ad libitum to ensure adequate hydration. The ewes were randomly allocated into four homogeneous groups of 10 animals each: control group (C), UDCA group (UDCA), probiotic group (PRO) and UDCA + probiotic group (UDCA + Pro) (Figure 1). The control group received no supplementation. The UDCA group was supplemented daily with 5 mg/kg of UDCA administered orally until parturition, calculated on the basis of body weight. The PRO group was given 0.2 mL/kg of probiotic formulation (Rebiotic Rumiflor, Reva Nutrition) calculated according to body weight until the time of birth via oral gavage according to the user instructions (Table 2). The UDCA + Pro group received both supplements at the specified doses. The dosage regimen of UDCA was formulated on the basis of protocols and experimental evidence reported in previous studies (Fan et al. 2025).

**TABLE 1:** Dietary nutrient content of late‐gestation sheep and composition of the sheep dairy concentrate feed.

Item	Value
**Ingredients (g/day as‐fed basis)**	
Sheep dairy concentrate feed	850
Corn silage	500
Wheat straw	500
Alfalfa hay	350
Cracked corn	100
**Dry matter content of ingredients (%)**	
Sheep dairy concentrate feed	88.5
Corn silage	25.0
Wheat straw	85.0
Alfalfa hay	90.0
Cracked corn	88.0
Total dry matter intake (g/day)	1705
**Nutrient composition of total ration (DM basis)**	
Crude protein (%)	14.0
Neutral detergent fibre (NDF, %)	37.35
Acid detergent fibre (ADF, %)	21.9
Crude ash (%)	6.9
Fat (%)	3.0
Non‐fibre carbohydrate (NFC, %)	38.75
Metabolizable energy (Mcal/kg DM)	2.45
**Composition of sheep dairy concentrate feed (% of DM)**	
Barley	58.0
Molasses	4.3
Corn	14.0
Sunflower meal (28% CP)	20.0
DCP	0.2
Marble powder	2.6
Salt	0.8
Vitamin–mineral mixture	0.1

Abbreviations: CFU, colony‐forming unit; DM, dry matter.

**TABLE 2:** Probiotic composition.

Microorganism	CFU/mL
*Enterococcus faecium* CCM 6226	1 × 10^7^
*Lactobacillus brevis* DSM 12835	1 × 10^6^
*Lactobacillus buchneri* CCM 1819	1 × 10^6^
*Lactobacillus Fermentum* NCIMB 41636	1 × 10^7^
*Lactobacillus paracasei* DSM 16245	1 × 10^7^
*Lactobacillus plantarum* C KKP/778/p	1 × 10^6^
*Lactobacillus plantarum* DSM 12836	1 × 10^6^
*Pediococcus acidilactici* DSM 16243	1 × 10^6^
*Pediococcus acidilactici* CNCM MA 18/5 M (DSM 11673)	1 × 10^6^
*Pediococcus pentosaceus* NCIMB 30168	1 × 10^6^
*Sacchoromyces cerevisiae* IFO 0203	1 × 10^7^
*Bacillus subtilis* C‐3102 (DSM 15544)	1 × 10^7^
*Enterococcus faecium* NCIMB 10415	1 × 10^6^
*Lactobacillus plantarum* DSM 29024	1 × 10^6^
*Lactobacillus plantarum* LP287 DSM 5257 ATCC 55058	1 × 10^6^
*Lactobacillus plantarum* DSMZ 16627	1 × 10^6^
*Lactobacillus plantarum* NCIMB 30083	1 × 10^6^

### 2.3 Health Monitoring and Sampling

The newborn lambs’ health status and clinical parameters were recorded, along with their birth weights (Figure 1). Blood samples were collected from the jugular vein of ewes before the initiation of the experimental treatments and immediately after parturition. For the lambs, blood samples were collected at 24 h postpartum and centrifuged (3000 *g*, 10 min, 4°C). The resulting sera were stored at −80°C until further analysis. Colostrum samples were collected from the ewes immediately following parturition, and lambs were bottle‐fed colostrum at 10% of their body weight within the first 24 h (with at least half provided within the first 6 h). Initial colostrum samples were stored at −80°C until analysis. The health status of lambs was monitored daily by a veterinarian. Lambs were weighed four times during the suckling period (D0, D2, D30 and D60) to monitor daily weight gain. The experiment concluded at 60 days postpartum with the weaning of the lambs (Figure 1). In the initial 0–10‐day period following birth, the primary source of nutrition for lambs was breast milk. During the subsequent 60‐day period, a feeding programme comprising milk, lamb starter feed and roughage (dry alfalfa hay) was implemented. The quantity of lamb starter feed, which was initially administered at a rate of 50–100 g, was increased to 300–350 g by the 60th day. The intake of roughage commenced at 50 g/day during the initial stages and reached 200 g/day by the 60th day. Throughout this period, a free‐choice system was employed, whereby the lambs were permitted to suckle their mothers at any time. Furthermore, clean water was consistently accessible to the lambs. The composition of the lamb starter feed provided to the lambs is outlined in Table 3, and net energy for maintenance (NE_m_) and gain (NE_g_) values are calculated according to National Research Council guidelines (NRC 2007).

**TABLE 3:** composition of the lamb starter feed.

Nutrient	Content
Dry matter (%)	88.00
Crude protein (%)	18.00
Metabolizable energy (Mcal/kg)	2.80
Net energy maintenance (NE_m_, Mcal/kg)	2.98
Net energy gain (NE_g_, Mcal/kg)	2.88
Calcium (Ca, %)	1.00
Phosphorus (P, %)	0.50
Sodium (Na, %)	0.25
Crude fibre (%)	11.00
Crude ash (%)	8.00
Vitamin A (IU/kg)	15.000
Vitamin D3 (IU/kg)	5.000
Vitamin E (mg/kg)	25

### 2.4 Serum and Colostrum IgG, Serum TP, Albumin, Total Antioxidant Capacity (TAC) and Total Oxidant Status (TOS) Analyses

Serum TP and albumin concentrations were measured using an autoanalyser (BT 3000 plus) and commercial kits. IgG concentrations in serum samples from both ewes and lambs, as well as in colostrum samples from ewes, were determined using the Sheep Immunoglobulin ELISA kit (BT Lab, Bioassay Technology Lab, Zhejiang, China, Cat No.: E0019Sh) according to the manufacturer's instructions. The absorbance values were measured using the BioTek Epoch 2 microplate reader, and each sample was measured in triplicate to ensure accuracy and reliability. To assess the antioxidant defence capacity, TAC was measured, whereas TOS was evaluated to determine the increase in oxidative stress (Bulut et al. 2023). TAC and TOS in serum samples were evaluated using ELISA kits (Elabscience Biotechnology Co. Ltd., USA, Cat No.: E‐BC‐K801 (TAC), E‐BC‐K802 (TOS)) according to the manufacturer's protocol. Serum TAC and TOS measurements were conducted using a colorimetric method, and the results were used to assess oxidative balance in the plasma (Dik et al. 2014; Gökçe et al. 2022; Sales et al. 2018).

### 2.5 Statistical Analysis

Prior to all statistical analyses, the assumptions of normality and homogeneity of variances were evaluated for each biochemical and immunological parameter. Normality of residuals was assessed using the Shapiro–Wilk test (*p* > 0.05 indicating normal distribution), and homogeneity of variances was tested using Levene's test (Tables S1 and S2). When Levene's test indicated significant variance heterogeneity (*p* < 0.05), logarithmic transformation (log_10_) was applied to stabilize variance, and Levene's test was repeated to assess improvement. Parameters that met the assumptions in either their original or transformed form were analysed using a Mixed Linear Model (MLM), in which group and time were entered as fixed effects, and subject ID was modelled as a random effect to account for repeated measures.

For variables violating normality or homoscedasticity assumptions even after transformation, a generalized linear model (GLM) was employed using a gamma distribution and a log link function, appropriate for non‐normally distributed, heteroscedastic data. In cases where only a single measurement per group was available, a one‐way ANOVA was conducted. When homogeneity of variances was met, Tukey's HSD test was used for post hoc comparisons; otherwise, Games–Howell post hoc test was applied. For data with only two time points within subjects, paired‐sample *t*‐tests were conducted.

Survival data were analysed using the Kaplan–Meier estimator, and survival distributions across treatment groups were compared using the log‐rank (Mantel–Cox) test. Additionally, the log‐rank test for trend was employed to assess ordinal trends in survival, and the Gehan–Breslow–Wilcoxon test was used to emphasize early survival differences.

To investigate the influence of maternal and colostral parameters on neonatal serum IgG concentrations, multiple linear regression analyses were conducted. Prior to modelling, the assumptions of linear regression—normality of residuals and homoscedasticity—were verified using the Shapiro–Wilk test and standardized residual plots, respectively. Two regression models were constructed: The first model included maternal IgG, maternal TP and their interaction; the second model incorporated colostrum IgG along with the three‐way interaction among maternal IgG, maternal TP and colostrum IgG. All predictors were mean‐centred before computing interaction terms to reduce multicollinearity. Models were fitted using the enter method, and significance was set at *p *< 0.05.

All statistical analyses were performed using IBM SPSS Statistics (Version 26) for general, regression and post hoc procedures, whereas GraphPad Prism (Version v10.4.2.) was used specifically for survival analyses and visualization.

## 3 Results

### 3.1 Live Weight Gain and Birth Statistics in Neonatal Lambs

**FIGURE 2:**
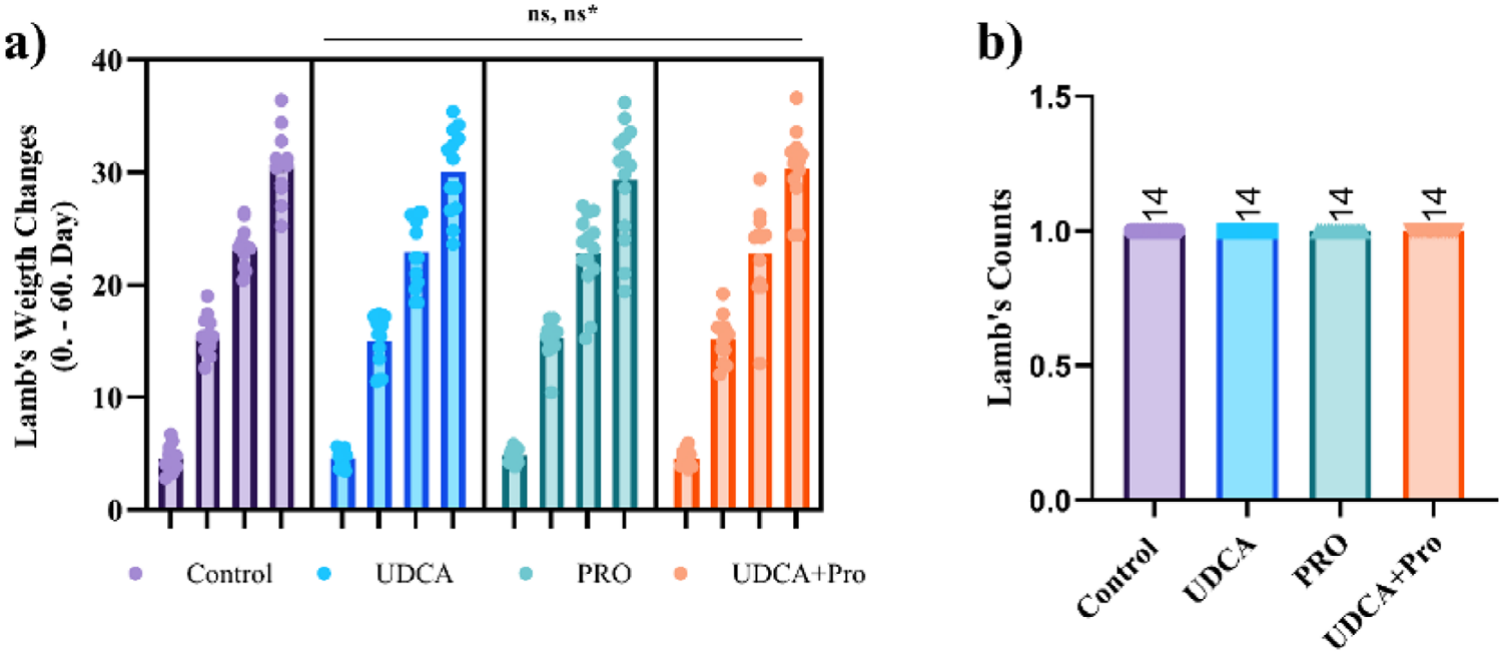
(a) Changes in live weight of lambs from birth to Day 60 across and within treatment groups. (b) Number of lambs born per group. Treatment groups: C = control, UDCA = ursodeoxycholic acid, PRO = probiotic, UDCA + PRO = combination of ursodeoxycholic acid and probiotic. The notation ‘ns’ indicates no statistically significant time‐dependent differences in live weight between groups, whereas ‘ns*’ denotes no significant within‐group differences across time points (Days 0, 15, 30 and 60) (*p* > 0.05).

The live weight gain and survival outcomes of lambs born to ewes receiving different prepartum treatments were evaluated. Statistical analysis revealed no significant differences among the groups in terms of average live weight gain, number of lambs born per ewe or the distribution of singleton and twin births (*p* > 0.05) (Figure 2). Detailed data on birth type distribution are not shown.

**FIGURE 3:**
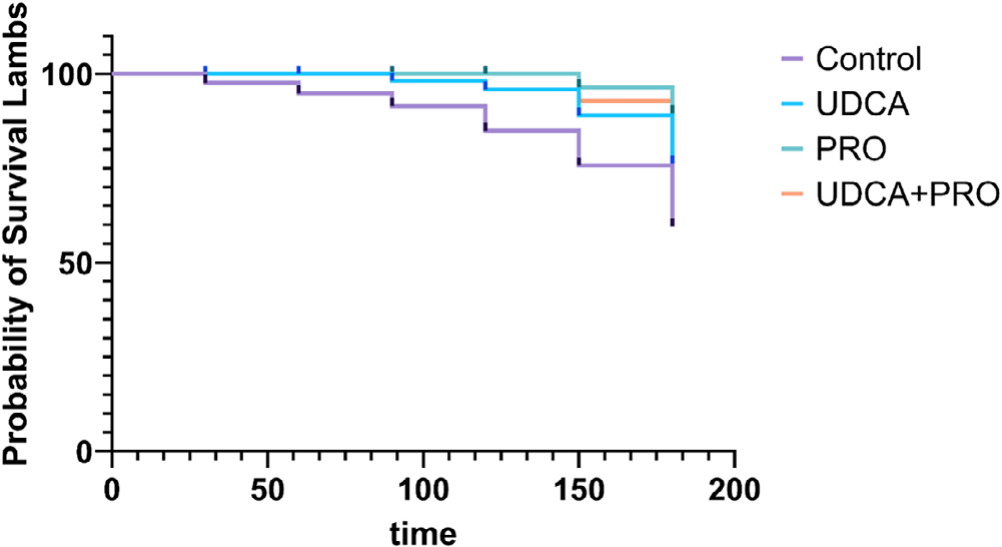
Kaplan–Meier survival curves of lambs born to ewes receiving different prepartum treatments. The groups include control (no supplementation), UDCA (ursodeoxycholic acid), PRO (probiotic) and UDCA + PRO. Survival probability was tracked over a 180‐day period.

### 3.2 Effects of Experimental Treatments and Sampling Time on Sheep Physiological Profiles

According to the Type III tests of fixed effects from the MLM, no significant group differences were observed for any of the biochemical parameters evaluated (all *p* > 0.05). However, several parameters demonstrated statistically significant time effects. Specifically, triglyceride (*F* = 28.950, *p *< 0.001), cholesterol (*F* = 13.237, *p* = 0.002), gamma‐glutamyl transferase (GGT) (*F* = 28.778, *p* < 0.001) and bilirubin (*F* = 10.867, *p* = 0.004) levels showed significant changes between the pre‐ and postpartum periods across all groups. In contrast, no significant group × time interaction effects were detected for any of the parameters (all *p* > 0.05), indicating that the observed time‐related changes were consistent across treatment groups (Table 4). The GLM analysis revealed that glucose (Wald *χ* ^2^ = 55.02, *p* < 0.001) and TAC (Wald *χ* ^2^ = 65.175, *p* < 0.001) levels significantly changed over time, regardless of group allocation. Additionally, a significant group effect was observed for TOS (Wald *χ* ^2^ = 18.16, *p* < 0.001), and a significant group × time interaction was also detected for both TOS (Wald *χ* ^2^ = 15.59, *p* < 0.001) and TAC (Wald *χ* ^2^ = 9.644, *p* = 0.022), indicating that the changes over time in these parameters varied by treatment group. No statistically significant group, time or interaction effects were detected for alkaline phosphatase (ALP), alanine aminotransferase (ALT) or TP (*p* > 0.05 for all), nor were any group‐based or interaction effects noted for glucose and TAC despite their significant time main effects (Table 5).

**TABLE 4:** Type III tests of fixed effects from the mixed linear model (MLM) for sheep physiological parameters: effects of group, time and group × time interaction.

Type III tests of fixed effects (MLM)			
	Effect	*F*	*p* value
Aspartate aminotransferase (U/L)	Groups	1.157	0.357
	Times	2.704	0.120
	Groups × Times	2.044	0.148
Creatinine (µmol/L)	Groups	0.308	0.819
	Times	2.336	0.146
	Groups × Times	2.183	0.130
Triglycerides (mmol/L)	Groups	0.850	0.487
	Times	28.950	<0.001*
	Groups × Times	1.739	0.199
Cholesterol (mmol/L)	Groups	0.440	0.728
	Times	13.237	0.002
	Groups × Times	2.477	0.099
Gamma‐glutamyl transferase (U/L)	Groups	0.997	0.419
	Times	28.778	<0.001*
	Groups × Times	1.015	0.412
Total bilirubin (µmol/L)	Groups	0.252	0.85
	Times	10.867	0.004
	Groups × Times	0.899	0.46
Albumin (g/dL)	Groups	0.64	0.60
	Times	0.74	0.40
	Groups × Times	2.72	0.08
Blood urea nitrogen (mmol/L)	Groups	1.130	0.367
	Times	1.166	0.296
	Groups × Times	0.731	0.548
Immunoglobulin G (mg/mL)	Groups	1.310	0.305
	Times	2.525	0.132
	Groups × Times	0.239	0.868

Note: The asterisk (*) indicates statistically significant differences at the *p* < 0.05 level.

**TABLE 5:** Generalized linear model analysis of sheep physiological parameters: group, time and group × time interaction effects.

Tests of model effects (GLM)			
	Effect	Wald chi‐square	*p* value
Glucose (mmol/L)	Groups	0.47	0.93
	Times	55.02	<0.001*
	Groups × Times	4.08	0.25
Alkaline phosphatase (U/L)	Groups	4.186	0.242
	Times	0.672	0.412
	Groups × Times	1.728	0.630
Total protein (g/L)	Groups	1.05	0.79
	Times	0.07	0.79
	Groups × Times	6.02	0.11
Alanine aminotransferase (U/L)	Groups	4.35	0.23
	Times	0.81	0.37
	Groups × Times	2.02	0.57
Total oxidant status (µmol H_2_O_2_ eq/L)	Groups	18.16	<0.001
	Times	0.89	0.34
	Groups × Times	15.59	<0.001*
Total antioxidant capacity (mM Trolox)	Groups	5.487	0.139
	Times	65.175	0.000
	Groups × Times	9.644	0.022

*Note*: The asterisk (*) indicates statistically significant differences at the *p* < 0.05 level.

### 3.3 Comparative Analysis of Physiological Profiles Among Treatment Groups Over Time

Across all parameters, including aspartate aminotransferase (AST), CREA, triglyceride, cholesterol, GGT, bilirubin, albumin, blood urea nitrogen (BUN), glucose, ALP, TP, ALT, TOS, TAC and IgG, no statistically significant differences were observed between the groups in either the prepartum or postpartum periods (adjusted *p* > 0.05 for all comparisons). Although a few comparisons showed trends toward significance (e.g., control vs. PRO for AST in the prepartum period, *p* = 0.1018), these did not reach statistical significance. Notably, for TOS levels in the prepartum period, there were statistically significant differences between the control and PRO groups (*p* = 0.0004), UDCA and PRO (*p* = 0.0003) and PRO versus UDCA + PRO (*p* = 0.022). However, these differences were not observed in the postpartum measurements. Overall, no consistent or robust group‐based differences in biochemical parameters were detected across time points, except for the observed TOS changes in the prepartum phase (Table S3).

### 3.4 Within‐Group Comparisons of Biochemical Parameters Between Pre‐ and Postpartum Periods

Paired‐sample *t*‐test analyses revealed significant intragroup differences between the pre‐ and postpartum periods for several biochemical parameters. In the control group, glucose levels significantly decreased postpartum compared to prepartum (*p* = 0.024), with a mean difference of −0.3951. Similarly, triglyceride concentrations increased significantly (mean difference = 0.3433, *p* = 0.01), whereas creatinine levels showed a marginally significant decrease (*p* = 0.053). In the UDCA‐treated group, glucose and triglyceride levels differed significantly between time points, with respective mean differences of −0.3313 (*p* = 0.015) and 0.3161 (*p* = 0.015). Moreover, a significant reduction in IgG levels was observed postpartum (mean difference = −1.882, *p* = 0.019). Within the PRO group, GGT activity decreased significantly from prepartum to postpartum (mean difference = −14.8, *p* = 0.021), accompanied by significant increases in TOS and TAC, with mean differences of 54.694 (*p* = 0.032) and 0.134 (*p* = 0.039), respectively. In addition, a significant decrease in IgG levels was detected (mean difference = −1.042, *p* = 0.043). In the combined UDCA + PRO group, glucose levels significantly decreased postpartum (mean difference = −0.3991, *p* = 0.031). GGT activity and cholesterol concentrations also declined (mean differences = −13.4 and −19.8; *p* = 0.033 and *p* = 0.013, respectively). Furthermore, significant increases were observed in TOS (mean difference = 4.79, *p* = 0.034) and TAC (mean difference = 0.216, *p* = 0.005). IgG levels in this group also decreased significantly in the postpartum period (mean difference = −0.764, *p* = 0.037) (Table 6).

**TABLE 6:** Prepartum vs. postpartum physiological changes within treatment groups.

	**Mean**	**Std. error mean**	**Sig. (2‐tailed)**	
**Control (prepartum–postpartum)**				
Aspartate aminotransferase	0.03384	0.05875	0.595	ns
Glucose	−0.39510	0.11190	0.024	*
Creatinine	−0.23800	0.08726	0.053	*
Triglyceride	0.34330	0.07419	0.010	*
Gamma‐glutamyl transferase	−8.80000	4.49889	0.122	ns
Total bilirubin	0.07400	0.03750	0.120	ns
Albumin	0.01558	0.05803	0.802	ns
Total protein	−0.02930	0.04533	0.553	ns
Alanine aminotransferase	−0.11344	0.12052	0.400	ns
Blood urea nitrogen	−2.60000	2.01494	0.266	ns
Cholesterol	−10.00000	6.76018	0.21316	ns
Alkaline phosphatase	0.01020	0.09430	0.91907	ns
Total oxidant status	0.58600	2.97251	0.853	ns
Total antioxidant capacity	1.44200	0.73721	0.122	ns
Immunoglobulin G	−0.40400	0.32113	0.277	ns
**UDCA (prepartum–postpartum)**				
Aspartate aminotransferase	0.01949	0.09652	0.850	ns
Glucose	−0.33133	0.08156	0.015	*
Creatinine	−0.06200	0.11508	0.619	ns
Triglyceride	0.31609	0.07757	0.015	*
Gamma‐glutamyl transferase	−22.20000	8.23043	0.054	*
Total bilirubin	0.02000	0.03592	0.607	ns
Albumin	0.00509	0.05864	0.935	ns
Total protein	−0.01779	0.05679	0.770	ns
Alanine aminotransferase	0.10754	0.12182	0.427	ns
Blood urea nitrogen	−2.80000	2.15407	0.263	ns
Cholesterol	−8.80000	5.37959	0.177	ns
Alkaline phosphatase	0.03060	0.07439	0.702	ns
Total oxidant status	−2.70600	5.24757	0.633	ns
Total antioxidant capacity	0.07600	0.03995	0.13	ns
Immunoglobulin G	−1.88200	0.49738	0.019	*
**PRO (prepartum–postpartum)**				
Aspartate aminotransferase	0.21954	0.08729	0.066	ns
Glucose	−0.17729	0.12975	0.244	ns
Creatinine	0.06400	0.04707	0.246	ns
Triglyceride	0.19819	0.09626	0.109	ns
Gamma‐glutamyl transferase	−14.80000	4.02989	0.021	*
Total bilirubin	0.09200	0.03891	0.077	ns
Albumin	0.14798	0.06238	0.077	ns
Total protein	0.10934	0.06696	0.178	ns
Alanine aminotransferase	−0.04410	0.18599	0.824	ns
Blood urea nitrogen	0.00000	1.92354	1.000	ns
Cholesterol	0.40000	3.69594	0.919	ns
Alkaline phosphatase	−0.06036	0.09071	0.542	ns
Total oxidant status	54.694	22.46603	0.032	*
Total antioxidant capacity	0.134	0.04434	0.039	*
Immunoglobulin G	−1.042	0.57255	0.043	*
**UDCA** + **PRO (prepartum–postpartum)**				
Aspartate aminotransferase (U/L)	−0.02465	0.04897	0.641	ns
Glucose (mmol/L)	−0.39914	0.12269	0.031	*
Creatinine (µmol/L)	−0.02600	0.07941	0.760	ns
Triglyceride (mmol/L)	0.09058	0.10132	0.422	ns
Gamma‐glutamyl transferase (U/L)	−13.40000	4.19047	0.033	*
Total bilirubin (µmol/L)	0.04000	0.02214	0.145	ns
Albumin (g/L)	−0.07294	0.04043	0.146	ns
Total protein (g/L)	−0.09165	0.03944	0.081	ns
Alanine aminotransferase (U/L)	−0.21743	0.10342	0.103	ns
Blood urea nitrogen (mmol/L)	0.80000	2.39583	0.755	ns
Cholesterol (mmol/L)	−19.80000	4.68402	0.013	*
Alkaline phosphatase (U/L)	−0.19622	0.19210	0.365	ns
Total oxidant status (µmol H_2_O_2_ eq/L)	4.79000	3.42461	0.034	*
Total antioxidant capacity (mmol Trolox eq/L)	0.21600	0.03750	0.005	*
Immunoglobulin G (mg/mL)	−0.76400	2.44002	0.037	*

*Note*: The asterisk (*) indicates statistically significant differences at the *p* < 0.05 level, whereas ‘ns’ denotes non‐significant differences (*p* ≥ 0.05).

### 3.5 Serum‐Based Assessment of Physiological Parameters in Lambs

One‐way ANOVA was performed to assess differences in serum biochemical parameters among treatment groups in lambs. Statistically significant group effects were observed for IgG (*p* = 0.037), TP (*p* = 0.021) and albumin (*p* = 0.011), indicating that these parameters varied significantly among the four experimental groups (control, UDCA, PRO and UDCA + PRO). Specifically, lambs in the UDCA + PRO group exhibited the highest IgG levels (15.22 ± 3.08 mg/mL), compared to the control group (11.92 ± 1.58 mg/mL). Similarly, TP concentrations were elevated in the PRO (66.85 ± 11.10 g/L) and UDCA (65.72 ± 15.15 g/L) groups relative to the control (53.43 ± 18.86 g/L). Albumin concentrations were also higher in the PRO (25.17 ± 3.60 g/L) and UDCA (24.50 ± 3.73 g/L) groups compared to the control (20.50 ± 4.97 g/L). In contrast, TOS and TAC levels did not differ significantly between groups (*p* = 0.46 and *p* = 0.93, respectively) (Table 7). No statistically significant differences were observed among the other biochemical parameters (AST, ALT, ALP, GGT, bilirubin, cholesterol, triglycerides, glucose, BUN and creatinine) across treatment groups (data not shown).

**TABLE 7:** Serum levels of total oxidant status (TOS), total antioxidant capacity (TAC), immunoglobulin G (IgG), total protein and albumin in lambs across experimental groups.

		*n*	Mean	Std. dev.	Std. error	*p*
Total oxidant status (µmol H_2_O_2_ eq/L)	Control	6	12.7617	4.77314	1.94863	0.46
	UDCA	6	17.1217	5.42840	2.21614	
	PRO	6	14.4983	4.00495	1.63502	
	UDCA + PRO	6	14.2250	5.96289	2.43434	
Total antioxidant capacity (mmol Trolox eq/L)	Control	6	1.2700	0.06542	0.02671	0.93
	UDCA	6	1.2767	0.05279	0.02155	
	PRO	6	1.2767	0.08335	0.03403	
	UDCA + PRO	6	1.2467	0.12596	0.05142	
Immunoglobulin G (mg/mL)	Control	6	11.9233	1.57997	0.64502	0.037*
	UDCA	6	13.5850	1.24829	0.50961	
	PRO	6	13.7433	1.69059	0.69018	
	UDCA + PRO	6	15.2233	3.08392	1.25900	
Total protein (g/L)	Control	6	53.4333	18.85510	7.69756	0.021*
	UDCA	6	65.7167	15.15327	6.18630	
	PRO	6	66.8500	11.09842	4.53091	
	UDCA + PRO	6	54.4500	9.41377	3.84315	
Albumin (g/L)	Control	6	20.5000	4.96991	2.02896	0.011*
	UDCA	6	24.5000	3.72827	1.52206	
	PRO	6	25.1667	3.60093	1.47007	
	UDCA + PRO	6	21.0000	4.04969	1.65328	

*Note*: The asterisk (*) indicates statistically significant differences at the *p* < 0.05 level.

Abbreviation: UDCA, ursodeoxycholic acid.

### 3.6 Colostrum Quality Parameters: TAC, TOS, IgG and Nutrient Composition

One‐way ANOVA revealed statistically significant group differences for colostral IgG (*p* = 0.017), fat content (*p* = 0.010) and lactose concentration (*p* = 0.050), indicating that maternal treatment influenced the composition of colostrum. IgG concentrations were highest in the UDCA + PRO group (1.28 ± 0.18 mg/mL), followed by the PRO (1.14 ± 0.06 mg/mL) and UDCA (1.15 ± 0.06 mg/mL) groups, compared to the control group (1.01 ± 0.03 mg/mL). Fat content was significantly elevated in the UDCA (13.13 ± 1.95) and PRO (12.93 ± 2.29) groups relative to the control group (9.78 ± 3.76). Similarly, lactose levels were higher in the UDCA (13.97 ± 0.84) and PRO (13.90 ± 2.18) groups compared to the control (11.35% ± 1.59%). No statistically significant differences were observed for TOS, TAC, solids‐not‐fat (SNF), density, freezing point, salt content, pH, conductivity or protein content (*p* > 0.05 for all) (Table 8).

**TABLE 8:** Group‐based comparison of colostral total antioxidant capacity, total oxidant status, immunoglobulin G and nutritional composition.

		*n*	Mean	Std. dev.	Std. error	*p*
Total oxidant status (µmol H_2_O_2_ eq/L)	Control	6	66.2080	15.38485	6.88031	0.245
	UDCA	6	57.0220	14.37694	6.42956	
	PRO	6	42.8500	7.25692	3.62846	
	UDCA + PRO	6	77.7700	48.10254	21.51211	
Total antioxidant capacity (mmol Trolox eq/L)	Control	6	0.3800	0.25729	0.11507	0.71
	UDCA	6	0.4700	0.13874	0.06205	
	PRO	6	0.5125	0.04924	0.02462	
	UDCA + PRO	6	0.4200	0.18561	0.08301	
Immunoglobulin G (mg/mL)	Control	6	1.0062	0.06797	0.03040	0.017*
	UDCA	6	1.1500	0.05831	0.02608	
	PRO	6	1.1375	0.05620	0.02810	
	UDCA + PRO	6	1.2800	0.17621	0.07880	
Solids‐not‐fat (SNF)	Control	6	25.4833	1.22216	0.49894	0.684
	UDCA	6	22.1333	5.99021	2.44549	
	PRO	6	23.3000	6.13254	2.50360	
	UDCA + PRO	6	22.1500	5.75804	2.35071	
Density	Control	6	84.3833	7.01211	2.86268	0.664
	UDCA	6	71.0500	24.38744	9.95613	
	PRO	6	76.9000	23.66880	9.66275	
	UDCA + PRO	6	73.1500	20.27913	8.27892	
Freezing point	Control	6	−2.3757	0.24165	0.09865	0.588
	UDCA	6	−2.0317	0.65591	0.26777	
	PRO	6	−2.1100	0.67770	0.27667	
	UDCA + PRO	6	−1.9472	0.63805	0.26048	
Salt	Control	6	2.0333	0.12111	0.04944	0.629
	UDCA	6	1.7333	0.50067	0.20440	
	PRO	6	1.8500	0.50892	0.20777	
	UDCA + PRO	6	1.7333	0.46332	0.18915	
pH	Control	6	10.8333	0.05164	0.02108	0.683
	UDCA	6	10.8333	0.05164	0.02108	
	PRO	6	10.8667	0.05164	0.02108	
	UDCA + PRO	6	10.8333	0.05164	0.02108	
Conductivity	Control	6	4.2333	0.46332	0.18915	0.133
	UDCA	6	4.2333	0.12111	0.04944	
	PRO	6	4.5000	0.14142	0.05774	
	UDCA + PRO	6	4.5833	0.17224	0.07032	
Fat	Control	6	9.7833	3.76001	1.53502	0.010*
	UDCA	6	13.1250	1.95237	0.79705	
	PRO	6	12.9333	2.28794	0.93405	
	UDCA + PRO	6	11.8000	1.13137	0.46188	
Protein	Control	6	8.4000	0.61644	0.25166	0.244
	UDCA	6	9.3000	0.53666	0.21909	
	PRO	6	9.4000	1.16103	0.47399	
	UDCA + PRO	6	8.6500	1.34127	0.54757	
Lactose	Control	6	11.3500	1.59217	0.65000	0.043*
	UDCA	6	13.9667	0.84063	0.34319	
	PRO	6	13.9000	2.18449	0.89181	
	UDCA + PRO	6	13.0167	2.03216	0.82963	

*Note*: The asterisk (*) indicates statistically significant differences at the *p* < 0.05 level.

Abbreviation: UDCA, ursodeoxycholic acid.

### 3.7 Survival Analysis

The survival analysis using the Kaplan–Meier method revealed a statistically significant difference in survival rates among the experimental groups (log‐rank test: *χ* ^2^ = 15.57, df = 3, *p* = 0.0014) (Figure 3). The log‐rank test for trend also showed a significant linear trend in survival improvement across groups (*χ* ^2^ = 10.79, df = 1, *p* = 0.0010), indicating a consistent survival benefit in treated animals. Additionally, the Gehan–Breslow–Wilcoxon test, which gives more weight to early events, confirmed these findings with an even stronger significance level (*χ* ^2^ = 21.42, df = 3, *p* < 0.0001). Collectively, these results indicate that the treatments had a significant positive impact on lamb survival, particularly in the early postnatal period.

### 3.8 Influence of Maternal and Colostral IgG and Protein on Neonatal Immune Status: A Regression Approach

Two hierarchical regression models were constructed to assess the influence of maternal and colostral immune parameters on lamb serum IgG levels (Table 9). The first model (Model 1) explained 48.3% of the variance in Lamb IgG (adjusted *R* ^2^ = 0.406, *p* = 0.004). Maternal IgG (*p* = 0.005) and maternal TP (*p* = 0.027) were both positively and significantly associated with lamb IgG, whereas their interaction term showed a significant negative effect (*p* = 0.030), suggesting a moderation effect where the combined elevation of both predictors reduces their individual influence. In the second model (Model 2), colostrum IgG and the three‐way interaction term were added, improving the model's explained variance to 53.0% (adjusted *R* ^2^ = 0.431, *p* = 0.005). Both maternal IgG (*p* = 0.024) and colostrum IgG (*p* = 0.029) were significant positive predictors. The three‐way interaction term (maternal IgG × maternal TP × colostrum IgG) was marginally significant (*p* = 0.088) and negative, indicating that the synergistic effect of all three predictors may attenuate IgG transfer under certain biological conditions. Although not statistically significant at the conventional alpha level of 0.05, this marginal association suggests a potential trend that may become significant in larger samples or under more controlled biological conditions.

**TABLE 9:** Hierarchical regression models predicting lamb serum immunoglobulin G (IgG) concentrations from maternal and colostral immune parameters.

Predictor	*B*	Beta	*p* value
**Model 1—Predictors of lamb IgG**			
Maternal IgG	2.717	2.193	0.005*
Maternal TP	0.723	3.596	0.027*
Maternal IgG × Maternal TP	−0.053	−3.294	0.030*
**Model 2—Extended model with colostrum IgG**			
Maternal IgG	1.800	1.453	0.024*
Maternal TP	0.457	2.272	0.079 (ns)
Colostrum IgG	22.205	1.321	0.029*
Maternal IgG × Maternal TP × Colostrum IgG	−0.029	−2.331	0.088 (marginal)

*Note*: The asterisk (*) indicates statistically significant differences at the *p* < 0.05 level, whereas ‘ns’ denotes non‐significant differences (*p* ≥ 0.05).

Abbreviation: TP, total protein.

## 4 Discussion

The final stages of pregnancy represent a critical physiological transition period for ewes, characterized not only by accelerated foetal growth but also by increasing metabolic demands and heightened exposure to environmental and managerial stressors. External factors, such as shearing, transport, dietary changes, restricted housing conditions and social stress, combined with the hormonal shifts inherent to late gestation, exert considerable pressure on maternal homeostasis (Wei 2023). These stressors are known to exert lasting effects not only on the dam but also on the developmental programming of the foetus (A. W Bell and Greenwood 2016; Wu et al. 2013; Yusof et al. 2023). In the present study, we comprehensively evaluated the effects of prenatal supplementation with UDCA, probiotics, and their combination during the final third of gestation. Key outcomes included maternal physiological status, colostrum quality, neonatal immune response, oxidative stress regulation and survival. The findings indicate that the combined intervention (UDCA + PRO) was particularly effective in enhancing immune‐related and survival parameters in lambs. These results underscore the potential value of holistic prenatal support strategies in optimizing both maternal resilience and neonatal vitality under the physiological and environmental pressures of late gestation.

The time‐dependent changes observed between the pre‐ and postpartum periods—including fluctuations in triglyceride, cholesterol, GGT, bilirubin, glucose and TAC levels—reflect the physiological metabolic remodelling that occurs as ewes transition from pregnancy to lactation. During this stage, the increasing energy demands are met with limited DMI, primarily due to reduced rumen capacity caused by the expanding uterus (Bell et al. 1987; Roche et al. 2000). The high glucose requirement for lactose synthesis in milk production further contributes to a predisposition toward hypoglycemia, which is physiologically compensated through enhanced lipolysis and proteolysis (Bernabucci et al. 2006; Knegsel et al. 2005). In our study, the absence of significant group‐based differences in systemic biochemical parameters suggests that the administered supplements had limited influence on fundamental energy balance and that the observed changes are largely driven by universal physiological adaptations shared across all groups.

Postpartum alterations in TOS and TAC were particularly notable in the groups receiving probiotic supplementation. These changes may reflect the ability of probiotics to reinforce intestinal epithelial integrity, reduce dysbiosis and stabilize mucosal immunity—ultimately creating a more favourable oxidative environment (Savvidou et al. 2023; Y. Wang et al. 2017). Similarly, UDCA has been shown to alleviate hepatic mitochondrial stress and modulate the immune microenvironment via activation of bile acid receptors, such as FXR and TGR5 (Dicks et al. 2024; H. Jia and Dong 2024; Zhang et al. 2023). Taken together, these findings provide field‐based evidence that the combination of UDCA and probiotics may exert synergistic effects, particularly by supporting hepatic function and enhancing systemic redox homeostasis during the physiologically demanding postpartum phase.

In ruminants, transplacental transfer of IgG does not occur during gestation; therefore, colostrum intake within the first hours postpartum serves as the sole and essential source of passive immunity for the neonate (Castro et al. 2011; Cervenak and Kacskovics 2009). In our study, individual administration of either UDCA or probiotics did not significantly increase colostral IgG levels; however, their combined use resulted in a marked enhancement. Although probiotics are known to stimulate mucosal immune responses—such as intestinal dendritic cell activation and IgA production—systemic IgG induction requires more complex immunological processes, including germinal centre activation and B‐cell proliferation (Chiofalo et al. 2004; Elolimy et al. 2022). UDCA may potentiate this response by creating an anti‐inflammatory and immunomodulatory intestinal microenvironment, thereby enhancing the colonization efficacy and immunostimulatory capacity of probiotics (Francini et al. 2025; Z. He et al. 2022; Liu, Li, et al. 2024; Liu, Wang, et al. 2024). These findings suggest that maternal immune modulation during late gestation is not only feasible but can also translate into improved colostral immunological quality.

Elevated levels of IgG, TP and albumin in neonatal serum further confirm that these maternal interventions supported effective passive immunity transfer. The pronounced increase in serum IgG observed in the UDCA + PRO group underscores a synergistic interaction between enhanced colostrum quality and efficient neonatal intestinal absorption. FcRn‐mediated IgG transcytosis peaks within the first 6–12 h after birth and is highly dependent on both the concentration of IgG and the integrity of the intestinal epithelium (Hurley and Theil 2011; Kacskovics 2004). Hence, maternal supplementation may have simultaneously optimized both the quality of colostrum and the absorptive capacity of the neonatal gut. Supporting this interpretation, survival analysis revealed a significant reduction in early postnatal mortality in the UDCA + PRO group. Given that neonatal losses are predominantly attributed to inadequate immune transfer, metabolic instability and increased susceptibility to infectious agents, this outcome holds both biological and economic relevance (Dwyer 2008; Nowak and Poindron 2006; Oyieng et al. 2025; Wilson et al. 2025).

Our multiple regression analysis revealed that maternal serum IgG and TP levels were independently and positively associated with neonatal serum IgG concentrations. However, the interaction term between these two variables was negatively significant, and in the extended model, the three‐way interaction (maternal IgG × TP × colostrum IgG) approached statistical significance. This suggests the presence of non‐linear biochemical regulation, a common feature in complex biological systems. These findings indicate that neonatal IgG absorption does not follow a simple linear dose–response relationship; rather, it is dynamically governed by threshold effects and the saturation kinetics of biological transport systems. Given the limited availability and saturable nature of FcRn receptors responsible for IgG transcytosis across the intestinal epithelium, excessive IgG loading may paradoxically reduce transport efficiency (Dalakas and Spaeth 2021; Ko et al. 2025; Lafrance‐Vanasse et al. 2025). Therefore, instead of adhering to a ‘more is better’ paradigm, the optimization of maternal and colostral IgG levels within physiological boundaries is essential. This result underscores that the effectiveness of passive immune transfer depends not merely on the absolute concentration of immunoglobulins but on a balanced approach aligned with the absorptive capacity of the neonatal gut.

Taken together, the findings of this study demonstrate that prenatal supplementation with UDCA and probiotics during late gestation enhances colostrum quality, facilitates more efficient passive immune transfer, improves neonatal serum IgG and protein profiles and ultimately contributes to increased early postnatal lamb survival. The data support the notion that the physiological impacts of prenatal stress can be modulated through targeted immunonutritional strategies. Moreover, the observed synergistic effects of combined supplementation and the recognition of the biological limits of immunoglobulin transport highlight the importance of precision‐based maternal interventions. Such approaches offer a promising foundation for developing perinatal management strategies aimed at improving flock health, productivity and overall sustainability in modern sheep production systems.

## 5 Limitations and Future Perspectives

Despite the promising findings of this study, several limitations should be acknowledged. First, the study was conducted with a relatively small sample size (*n* = 40 ewes), which may limit the generalizability of the results to broader populations or different sheep breeds. Additionally, the investigation focused primarily on short‐term outcomes—such as early postnatal survival and immediate immune responses—whereas long‐term effects, including post‐weaning growth performance, reproductive capacity and overall lifetime productivity of the lambs, remain unaddressed. Potential impacts of prenatal treatments on other economically relevant traits, such as carcass quality and meat yield, were also not explored.

Future research should aim to address these limitations by incorporating larger sample sizes and examining the effects of prenatal interventions across diverse breeds and under various environmental and management conditions. Longitudinal studies assessing the lasting impact of such interventions on lamb growth trajectories, reproductive performance and production efficiency would be particularly valuable. Moreover, investigating molecular markers related to prenatal stress, inflammation and immune regulation in both ewes and lambs could enhance our understanding of the underlying biological mechanisms responsible for the observed outcomes. Finally, the inclusion of cost–benefit analyses—especially regarding the ability of these interventions to mitigate challenges associated with twin pregnancies—would provide practical guidance for producers. By addressing these gaps, future research could expand upon the current findings and contribute to a more comprehensive understanding of the benefits and applications of prenatal support strategies in modern sheep production systems.

## Author Contributions

Motivation/Concept: Durmus Hatipoglu, M. Burak Ates and Fatih Aladag. Design: Durmus Hatipoglu, M. Burak Ates and Fatih Aladag. Control/Supervision: Durmus Hatipoglu, M. Burak Ates and Fatih Aladag. Data collection and processing: Durmus Hatipoglu, M. Burak Ates and Fatih Aladag. Analysis and interpretation: Durmus Hatipoglu, M. Burak Ates and Fatih Aladag. Literature review: Durmus Hatipoglu, M. Burak Ates and Fatih Aladag. Writing the article: Durmus Hatipoglu. Critical review: Durmus Hatipoglu, M. Burak Ates and Fatih Aladag.

## Funding

This work was supported by Selcuk University Scientific Research Projects Coordination Office (project no. 23401162).

## Conflicts of Interest

The authors declare no conflicts of interest.

## Supporting information




**Supporting File 1**: vms370859‐sup‐0001‐SuppMat.docx

## Data Availability

The data that support the findings of this study are available on request from the corresponding author.
