# Supplementary material for: Effects of Prenatal Ursodeoxycholic Acid and Probiotic Supplementation on Stress Parameters, Immune Response and Lamb Survival Rates in Ewes
Source: Vet Med Sci. 2026 Jul 29;12(5):e70859. doi: 10.1002/vms3.70859 (PMC13418863; doi:10.1002/vms3.70859)
Supplement: Supplementary file 1 — Supporting File 1: vms370859‐sup‐0001‐SuppMat.docx [file VMS3-12-e70859-s001.docx]

|  | Levene's Test of Equality of Error Variances | | | | After log transformation | | | |
| --- | --- | --- | --- | --- | --- | --- | --- | --- |
|  | Levene Statistic | df1 | df2 | Sig. | Levene Statistic | df1 | df2 | Sig. |
| AST | 2,405 | 7 | 32 | 0,042* | 1,527 | 7 | 32 | 0,194 |
| Glucose | 7,83 | 7 | 32 | 0,00001* | 3,149 | 7 | 32 | 0,012* |
| Crea | 2,007 | 7 | 32 | 0,085 | - | - | - | - |
| Trig | 3,049 | 7 | 32 | 0,014* | 1,042 | 7 | 32 | 0,422 |
| Cholest | 2,267 | 7 | 32 | 0,048* | 2,275 | 7 | 32 | 0,053 |
| ALP | 2,412 | 7 | 32 | 0,042* | 2,669 | 7 | 32 | 0,027* |
| GGT | 1,108 | 7 | 32 | 0,382 | - | - | - | - |
| Bilirubine | 2,176 | 7 | 32 | 0,063 | - | - | - | - |
| Albumine | 2,351 | 7 | 32 | 0,047* | 1,067 | 7 | 32 | 0,419 |
| TP | 3,499 | 7 | 32 | 0,007* | 0,749 | 7 | 32 | 0,635 |
| ALT | 2,496 | 7 | 32 | 0,036* | 1,664 | 7 | 32 | 0,168 |
| BUN | 1,45 | 7 | 32 | 0,22 | - | - | - | - |
| TOS (GLM) | 21,714 | 7 | 32 | 0,000* | 3,196 | 7 | 32 | 0,011* |
| TAC | 69,977 | 7 | 32 | 0,000* | 0,800 | 7 | 32 | 0,593 |
| IgG | 2,077 | 7 | 32 | 0,075 |  |  |  |  |
| LambTOS | 0,733 | 3 | 20 | 0,542 |  |  |  |  |
| LambTAC | 2,488 | 3 | 20 | 0,089 |  |  |  |  |
| LambIgG | 0,91 | 3 | 20 | 0,45 |  |  |  |  |
| Lambalbumin | ,302 | 3 | 20 | ,824 |  |  |  |  |
| LambTP | 1,546 | 3 | 20 | ,234 |  |  |  |  |
| CholosTOS | 2,64 | 3,00 | 15,00 | 0,09 |  |  |  |  |
| CholosTAC | 5,06 | 3,00 | 15,00 | 0,01* | 3,91 | 3,00 | 15,00 | 0,03* |
| CholosIgG | 4,86 | 3,00 | 15,00 | 0,01* | 4,53 | 3,00 | 15,00 | 0,02 * |
| Cholos Fat-free dry matter | 3,96 | 3,00 | 20,00 | 0,07 |  |  |  |  |
| Cholos Density | 3,67 | 3,00 | 20,00 | 0,15 |  |  |  |  |
| Cholos Freezing point | 2,97 | 3,00 | 20,00 | 0,06 |  |  |  |  |
| CholosSalt | 3,92 | 3,00 | 20,00 | 0,07 |  |  |  |  |
| CholospH | - | 3,00 | 20,00 | 1,00 |  |  |  |  |
| CholosCo | 7,25 | 3,00 | 20,00 | 0,07 |  |  |  |  |
| CholosFat | 2,443 | 3 | 20 | ,094 |  |  |  |  |
| CholosProtein | 2,472 | 3 | 20 | ,091 |  |  |  |  |
| Choloslactose | 1,536 | 3 | 20 | ,236 |  |  |  |  |
| (p-values < 0.05 are indicated with an asterisk [*], indicating a violation of the homoscedasticity assumption (i.e., the presence of heteroscedasticity). For parameters that initially violated the homoscedasticity assumption, a logarithmic transformation (log10) was applied to improve variance homogeneity. Post-transformation p-values are reported for applicable parameters. Parameters without post-transformation results had already satisfied the homoscedasticity assumption at baseline; parameters that remained heteroscedastic after transformation are also marked with an asterisk [*]. | | | | | | | | |

**Supplementary Table 1.** Levene’s Test of Equality of Error Variances Before and After Logarithmic Transformation for Each Biochemical Parameter.

**Supplementary Table 2. Normality Assessment of Standardized Residuals Using the Shapiro–Wilk Test**

| Tests of Normality | | | |
| --- | --- | --- | --- |
|  | Shapiro-Wilk | | |
|  | Statistic | df | Sig. |
| Standardized Residual for LogAST | 0,987 | 40 | 0,93 |
| Standardized Residual for LogGlucose | 0,965 | 40 | 0,254 |
| Standardized Residual for Crea | 0,985 | 40 | 0,872 |
| Standardized Residual for LogTrig | 0,972 | 40 | 0,423 |
| Standardized Residual for LogCholest | 0,962 | 40 | 0,204 |
| Standardized Residual for LogALP | 0,943 | 40 | 0,054 |
| Standardized Residual for GGT | 0,949 | 40 | 0,069 |
| Standardized Residual for Bilirubine | 0,946 | 40 | 0,055 |
| Standardized Residual for LogAlbumine | 0,987 | 40 | 0,916 |
| Standardized Residual for LogTotalprotein | 0,983 | 40 | 0,803 |
| Standardized Residual for LogALT | 0,981 | 40 | 0,742 |
| Standardized Residual for BUN | 0,977 | 40 | 0,565 |
| Standardized Residual for TOS | 0,980 | 40 | 0,698 |
| Standardized Residual for TAC | 0,962 | 40 | 0,191 |
| Standardized Residual for IgG | 0,839 | 40 | 0,000* |
| Standardized Residual for TOSLamb | 0,946 | 24 | 0,218 |
| Standardized Residual for TASLamb | 0,957 | 24 | 0,379 |
| Standardized Residual for IgGLamb | 0,909 | 24 | 0,034 |
| Standardized Residual for TPLamb | ,967 | 24 | ,605 |
| Standardized Residual for albumineLamb | ,968 | 24 | ,623 |
| Standardized Residual for TOScholostrum | 0,876 | 19 | 0,018 |
| Standardized Residual for TACcholostrum | 0,951 | 19 | 0,415 |
| Standardized Residual for LogIgGcholostrum | 0,896 | 19 | 0,062 |
| Standardized Residual for Fat-free dry matter | 0,924 | 24 | 0,073 |
| Standardized Residual for Density | 0,912 | 24 | 0,077 |
| Standardized Residual for Freezing point | 0,927 | 24 | 0,83 |
| Standardized Residual for Salt | 0,918 | 24 | 0,059 |
| Standardized Residual for pH | 0,789 | 24 | 0,000* |
| Standardized Residual for co | 0,938 | 24 | 0,151 |
| Standardized Residual for Fat | ,932 | 24 | ,108 |
| Standardized Residual for Cholostrumprotein | ,973 | 24 | ,745 |
| Standardized Residual for Cholostrumlactose | ,974 | 24 | ,764 |
| (An asterisk [*] indicates p-values < 0.05, representing a significant violation of the normality assumption for standardized residuals as assessed by the Shapiro-Wilk test. Normality was assumed for parameters with p-values > 0.05.) | | | |

**Supplementary Table 3**. Pairwise Group Comparisons of Sheep Biochemical Parameters at Each Time Point Using Bonferroni-Adjusted Post Hoc Tests

| **Bonferroni's multiple comparisons test** | | | | | | |
| --- | --- | --- | --- | --- | --- | --- |
| **Parameter** | **Time (days)** | **Comparison** | **Mean Diff.** | **95,00% CI of diff,SE** | **Adjusted p-value** | **Sig.** |
| Aspartate  Aminotransferase | Prepartum | Control vs. UDCA | -0,056 | -0,2927 to 0,1807 | >0,9999 | ns |
|  |  | Control vs. PRO | -0,212 | -0,4487 to 0,02471 | 0,1018 | ns |
|  |  | Control vs. UDCA+PRO | -0,014 | -0,2507 to 0,2227 | >0,9999 | ns |
|  |  | UDCA vs. PRO | -0,156 | -0,3927 to 0,08071 | 0,4383 | ns |
|  |  | UDCA vs. UDCA+PRO | 0,042 | -0,1947 to 0,2787 | >0,9999 | ns |
|  |  | PRO vs. UDCA+PRO | 0,198 | -0,03871 to 0,4347 | 0,1498 | ns |
|  | Postpartum | Control vs. UDCA | -0,07 | -0,3067 to 0,1667 | >0,9999 | ns |
|  |  | Control vs. PRO | -0,028 | -0,2647 to 0,2087 | >0,9999 | ns |
|  |  | Control vs. UDCA+PRO | -0,07 | -0,3067 to 0,1667 | >0,9999 | ns |
|  |  | UDCA vs. PRO | 0,042 | -0,1947 to 0,2787 | >0,9999 | ns |
|  |  | UDCA vs. UDCA+PRO | 0 | -0,2367 to 0,2367 | >0,9999 | ns |
|  |  | PRO vs. UDCA+PRO | -0,042 | -0,2787 to 0,1947 | >0,9999 | ns |
|  | | | | | | |
| Creatinine | Prepartum | Control vs. UDCA | -0,158 | -0,4392 to 0,1232 | 0,7432 | ns |
|  |  | Control vs. PRO | -0,162 | -0,4432 to 0,1192 | 0,6899 | ns |
|  |  | Control vs. UDCA+PRO | -0,122 | -0,4032 to 0,1592 | >0,9999 | ns |
|  |  | UDCA vs. PRO | -0,004 | -0,2852 to 0,2772 | >0,9999 | ns |
|  |  | UDCA vs. UDCA+PRO | 0,036 | -0,2452 to 0,3172 | >0,9999 | ns |
|  |  | PRO vs. UDCA+PRO | 0,04 | -0,2412 to 0,3212 | >0,9999 | ns |
|  | Postpartum | Control vs. UDCA | 0,018 | -0,2632 to 0,2992 | >0,9999 | ns |
|  |  | Control vs. PRO | 0,14 | -0,1412 to 0,4212 | >0,9999 | ns |
|  |  | Control vs. UDCA+PRO | 0,09 | -0,1912 to 0,3712 | >0,9999 | ns |
|  |  | UDCA vs. PRO | 0,122 | -0,1592 to 0,4032 | >0,9999 | ns |
|  |  | UDCA vs. UDCA+PRO | 0,072 | -0,2092 to 0,3532 | >0,9999 | ns |
|  |  | PRO vs. UDCA+PRO | -0,05 | -0,3312 to 0,2312 | >0,9999 | ns |
|  | | | | | | |
| Triglycerides | Prepartum | Control vs. UDCA | -0,048 | -0,3404 to 0,2444 | >0,9999 | ns |
|  |  | Control vs. PRO | 0,096 | -0,1964 to 0,3884 | >0,9999 | ns |
|  |  | Control vs. UDCA+PRO | 0,034 | -0,2584 to 0,3264 | >0,9999 | ns |
|  |  | UDCA vs. PRO | 0,144 | -0,1484 to 0,4364 | >0,9999 | ns |
|  |  | UDCA vs. UDCA+PRO | 0,082 | -0,2104 to 0,3744 | >0,9999 | ns |
|  |  | PRO vs. UDCA+PRO | -0,062 | -0,3544 to 0,2304 | >0,9999 | ns |
|  | Postpartum | Control vs. UDCA | -0,074 | -0,3664 to 0,2184 | >0,9999 | ns |
|  |  | Control vs. PRO | -0,046 | -0,3384 to 0,2464 | >0,9999 | ns |
|  |  | Control vs. UDCA+PRO | -0,218 | -0,5104 to 0,07436 | 0,2639 | ns |
|  |  | UDCA vs. PRO | 0,028 | -0,2644 to 0,3204 | >0,9999 | ns |
|  |  | UDCA vs. UDCA+PRO | -0,144 | -0,4364 to 0,1484 | >0,9999 | ns |
|  |  | PRO vs. UDCA+PRO | -0,172 | -0,4644 to 0,1204 | 0,6468 | ns |
|  | | | | | | |
| Cholesterol | Prepartum | Control vs. UDCA | -5,4 | -24,46 to 13,66 | >0,9999 | ns |
|  |  | Control vs. PRO | -5 | -24,06 to 14,06 | >0,9999 | ns |
|  |  | Control vs. UDCA+PRO | 6 | -13,06 to 25,06 | >0,9999 | ns |
|  |  | UDCA vs. PRO | 0,4 | -18,66 to 19,46 | >0,9999 | ns |
|  |  | UDCA vs. UDCA+PRO | 11,4 | -7,663 to 30,46 | 0,614 | ns |
|  |  | PRO vs. UDCA+PRO | 11 | -8,063 to 30,06 | 0,6866 | ns |
|  | Postpartum | Control vs. UDCA | -4,2 | -23,26 to 14,86 | >0,9999 | ns |
|  |  | Control vs. PRO | 5,4 | -13,66 to 24,46 | >0,9999 | ns |
|  |  | Control vs. UDCA+PRO | -3,8 | -22,86 to 15,26 | >0,9999 | ns |
|  |  | UDCA vs. PRO | 9,6 | -9,463 to 28,66 | 0,9981 | ns |
|  |  | UDCA vs. UDCA+PRO | 0,4 | -18,66 to 19,46 | >0,9999 | ns |
|  |  | PRO vs. UDCA+PRO | -9,2 | -28,26 to 9,863 | >0,9999 | ns |
|  | | | | | | |
| Gamma-Glutamyl  Transferase | Prepartum | Control vs. UDCA | -2,6 | -21,89 to 16,69 | >0,9999 | ns |
|  |  | Control vs. PRO | -1,8 | -21,09 to 17,49 | >0,9999 | ns |
|  |  | Control vs. UDCA+PRO | 0 | -19,29 to 19,29 | >0,9999 | ns |
|  |  | UDCA vs. PRO | 0,8 | -18,49 to 20,09 | >0,9999 | ns |
|  |  | UDCA vs. UDCA+PRO | 2,6 | -16,69 to 21,89 | >0,9999 | ns |
|  |  | PRO vs. UDCA+PRO | 1,8 | -17,49 to 21,09 | >0,9999 | ns |
|  | Postpartum | Control vs. UDCA | -16 | -35,29 to 3,287 | 0,1565 | ns |
|  |  | Control vs. PRO | -7,8 | -27,09 to 11,49 | >0,9999 | ns |
|  |  | Control vs. UDCA+PRO | -4,6 | -23,89 to 14,69 | >0,9999 | ns |
|  |  | UDCA vs. PRO | 8,2 | -11,09 to 27,49 | >0,9999 | ns |
|  |  | UDCA vs. UDCA+PRO | 11,4 | -7,887 to 30,69 | 0,6373 | ns |
|  |  | PRO vs. UDCA+PRO | 3,2 | -16,09 to 22,49 | >0,9999 | ns |
|  | | | | | | |
| Total Bilirubin | Prepartum | Control vs. UDCA | 0,006 | -0,1083 to 0,1203 | >0,9999 | ns |
|  |  | Control vs. PRO | -0,024 | -0,1383 to 0,09029 | >0,9999 | ns |
|  |  | Control vs. UDCA+PRO | 0,02 | -0,09429 to 0,1343 | >0,9999 | ns |
|  |  | UDCA vs. PRO | -0,03 | -0,1443 to 0,08429 | >0,9999 | ns |
|  |  | UDCA vs. UDCA+PRO | 0,014 | -0,1003 to 0,1283 | >0,9999 | ns |
|  |  | PRO vs. UDCA+PRO | 0,044 | -0,07029 to 0,1583 | >0,9999 | ns |
|  | Postpartum | Control vs. UDCA | -0,048 | -0,1623 to 0,06629 | >0,9999 | ns |
|  |  | Control vs. PRO | -0,006 | -0,1203 to 0,1083 | >0,9999 | ns |
|  |  | Control vs. UDCA+PRO | -0,014 | -0,1283 to 0,1003 | >0,9999 | ns |
|  |  | UDCA vs. PRO | 0,042 | -0,07229 to 0,1563 | >0,9999 | ns |
|  |  | UDCA vs. UDCA+PRO | 0,034 | -0,08029 to 0,1483 | >0,9999 | ns |
|  |  | PRO vs. UDCA+PRO | -0,008 | -0,1223 to 0,1063 | >0,9999 | ns |
|  | | | | | | |
| Albumine | Prepartum | Control vs. UDCA | -0,042 | -0,2277 to 0,1437 | >0,9999 | ns |
|  |  | Control vs. PRO | -0,14 | -0,3257 to 0,04568 | 0,2509 | ns |
|  |  | Control vs. UDCA+PRO | 0 | -0,1857 to 0,1857 | >0,9999 | ns |
|  |  | UDCA vs. PRO | -0,098 | -0,2837 to 0,08768 | 0,885 | ns |
|  |  | UDCA vs. UDCA+PRO | 0,042 | -0,1437 to 0,2277 | >0,9999 | ns |
|  |  | PRO vs. UDCA+PRO | 0,14 | -0,04568 to 0,3257 | 0,2509 | ns |
|  | Postpartum | Control vs. UDCA | -0,054 | -0,2397 to 0,1317 | >0,9999 | ns |
|  |  | Control vs. PRO | -0,01 | -0,1957 to 0,1757 | >0,9999 | ns |
|  |  | Control vs. UDCA+PRO | -0,088 | -0,2737 to 0,09768 | >0,9999 | ns |
|  |  | UDCA vs. PRO | 0,044 | -0,1417 to 0,2297 | >0,9999 | ns |
|  |  | UDCA vs. UDCA+PRO | -0,034 | -0,2197 to 0,1517 | >0,9999 | ns |
|  |  | PRO vs. UDCA+PRO | -0,078 | -0,2637 to 0,1077 | >0,9999 | ns |
|  | | | | | | |
| Blood Urea  Nitrogen | Prepartum | Control vs. UDCA | -1 | -7,068 to 5,068 | >0,9999 | ns |
|  |  | Control vs. PRO | -3,6 | -9,668 to 2,468 | 0,6298 | ns |
|  |  | Control vs. UDCA+PRO | -4,2 | -10,27 to 1,868 | 0,3624 | ns |
|  |  | UDCA vs. PRO | -2,6 | -8,668 to 3,468 | >0,9999 | ns |
|  |  | UDCA vs. UDCA+PRO | -3,2 | -9,268 to 2,868 | 0,8869 | ns |
|  |  | PRO vs. UDCA+PRO | -0,6 | -6,668 to 5,468 | >0,9999 | ns |
|  | Postpartum | Control vs. UDCA | -1,2 | -7,268 to 4,868 | >0,9999 | ns |
|  |  | Control vs. PRO | -1 | -7,068 to 5,068 | >0,9999 | ns |
|  |  | Control vs. UDCA+PRO | -0,8 | -6,868 to 5,268 | >0,9999 | ns |
|  |  | UDCA vs. PRO | 0,2 | -5,868 to 6,268 | >0,9999 | ns |
|  |  | UDCA vs. UDCA+PRO | 0,4 | -5,668 to 6,468 | >0,9999 | ns |
|  |  | PRO vs. UDCA+PRO | 0,2 | -5,868 to 6,268 | >0,9999 | ns |
|  | | | | | | |
| Glucose | Prepartum | Control vs. UDCA | -1 | -59,40 to 57,40 | >0,9999 | ns |
|  |  | Control vs. PRO | -10,2 | -68,60 to 48,20 | >0,9999 | ns |
|  |  | Control vs. UDCA+PRO | -2,2 | -60,60 to 56,20 | >0,9999 | ns |
|  |  | UDCA vs. PRO | -9,2 | -67,60 to 49,20 | >0,9999 | ns |
|  |  | UDCA vs. UDCA+PRO | -1,2 | -59,60 to 57,20 | >0,9999 | ns |
|  |  | PRO vs. UDCA+PRO | 8 | -50,40 to 66,40 | >0,9999 | ns |
|  | Postpartum | Control vs. UDCA | 18,6 | -39,80 to 77,00 | >0,9999 | ns |
|  |  | Control vs. PRO | 30,6 | -27,80 to 89,00 | 0,9023 | ns |
|  |  | Control vs. UDCA+PRO | 3,2 | -55,20 to 61,60 | >0,9999 | ns |
|  |  | UDCA vs. PRO | 12 | -46,40 to 70,40 | >0,9999 | ns |
|  |  | UDCA vs. UDCA+PRO | -15,4 | -73,80 to 43,00 | >0,9999 | ns |
|  |  | PRO vs. UDCA+PRO | -27,4 | -85,80 to 31,00 | >0,9999 | ns |
|  | | | | | | |
| Alkaline  phosphatase | Prepartum | Control vs. UDCA | -0,1 | -0,5268 to 0,3268 | >0,9999 | ns |
|  |  | Control vs. PRO | 0,13 | -0,2968 to 0,5568 | >0,9999 | ns |
|  |  | Control vs. UDCA+PRO | 0,166 | -0,2608 to 0,5928 | >0,9999 | ns |
|  |  | UDCA vs. PRO | 0,23 | -0,1968 to 0,6568 | 0,8364 | ns |
|  |  | UDCA vs. UDCA+PRO | 0,266 | -0,1608 to 0,6928 | 0,5351 | ns |
|  |  | PRO vs. UDCA+PRO | 0,036 | -0,3908 to 0,4628 | >0,9999 | ns |
|  | Postpartum | Control vs. UDCA | -0,076 | -0,5028 to 0,3508 | >0,9999 | ns |
|  |  | Control vs. PRO | 0,064 | -0,3628 to 0,4908 | >0,9999 | ns |
|  |  | Control vs. UDCA+PRO | -0,034 | -0,4608 to 0,3928 | >0,9999 | ns |
|  |  | UDCA vs. PRO | 0,14 | -0,2868 to 0,5668 | >0,9999 | ns |
|  |  | UDCA vs. UDCA+PRO | 0,042 | -0,3848 to 0,4688 | >0,9999 | ns |
|  |  | PRO vs. UDCA+PRO | -0,098 | -0,5248 to 0,3288 | >0,9999 | ns |
|  | | | | | | |
| Total Protein | Prepartum | Control vs. UDCA | -0,028 | -0,2162 to 0,1602 | >0,9999 | ns |
|  |  | Control vs. PRO | -0,114 | -0,3022 to 0,07415 | 0,5885 | ns |
|  |  | Control vs. UDCA+PRO | 0,008 | -0,1802 to 0,1962 | >0,9999 | ns |
|  |  | UDCA vs. PRO | -0,086 | -0,2742 to 0,1022 | >0,9999 | ns |
|  |  | UDCA vs. UDCA+PRO | 0,036 | -0,1522 to 0,2242 | >0,9999 | ns |
|  |  | PRO vs. UDCA+PRO | 0,122 | -0,06615 to 0,3102 | 0,4654 | ns |
|  | Postpartum | Control vs. UDCA | -0,016 | -0,2042 to 0,1722 | >0,9999 | ns |
|  |  | Control vs. PRO | 0,028 | -0,1602 to 0,2162 | >0,9999 | ns |
|  |  | Control vs. UDCA+PRO | -0,052 | -0,2402 to 0,1362 | >0,9999 | ns |
|  |  | UDCA vs. PRO | 0,044 | -0,1442 to 0,2322 | >0,9999 | ns |
|  |  | UDCA vs. UDCA+PRO | -0,036 | -0,2242 to 0,1522 | >0,9999 | ns |
|  |  | PRO vs. UDCA+PRO | -0,08 | -0,2682 to 0,1082 | >0,9999 | ns |
|  | | | | | | |
| Alanine  aminotransferase | Prepartum | Control vs. UDCA | -0,212 | -0,6355 to 0,2115 | >0,9999 | ns |
|  |  | Control vs. PRO | 0,098 | -0,3255 to 0,5215 | >0,9999 | ns |
|  |  | Control vs. UDCA+PRO | 0,086 | -0,3375 to 0,5095 | >0,9999 | ns |
|  |  | UDCA vs. PRO | 0,31 | -0,1135 to 0,7335 | 0,2864 | ns |
|  |  | UDCA vs. UDCA+PRO | 0,298 | -0,1255 to 0,7215 | 0,3388 | ns |
|  |  | PRO vs. UDCA+PRO | -0,012 | -0,4355 to 0,4115 | >0,9999 | ns |
|  | Postpartum | Control vs. UDCA | 0,008 | -0,4155 to 0,4315 | >0,9999 | ns |
|  |  | Control vs. PRO | 0,17 | -0,2535 to 0,5935 | >0,9999 | ns |
|  |  | Control vs. UDCA+PRO | -0,016 | -0,4395 to 0,4075 | >0,9999 | ns |
|  |  | UDCA vs. PRO | 0,162 | -0,2615 to 0,5855 | >0,9999 | ns |
|  |  | UDCA vs. UDCA+PRO | -0,024 | -0,4475 to 0,3995 | >0,9999 | ns |
|  |  | PRO vs. UDCA+PRO | -0,186 | -0,6095 to 0,2375 | >0,9999 | ns |
|  | | | | | | |
| Total oxidant  status | Prepartum | Control vs. UDCA | 0,01312 | -0,4550 to 0,4812 | >0,9999 | ns |
|  |  | Control vs. PRO | -0,7687 | -1,237 to -0,3006 | 0,0004 | *** |
|  |  | Control vs. UDCA+PRO | -0,2467 | -0,7149 to 0,2214 | 0,8881 | ns |
|  |  | UDCA vs. PRO | -0,7818 | -1,250 to -0,3137 | 0,0003 | *** |
|  |  | UDCA vs. UDCA+PRO | -0,2599 | -0,7280 to 0,2082 | 0,7698 | ns |
|  |  | PRO vs. UDCA+PRO | 0,5219 | 0,05381 to 0,9900 | 0,022 | * |
|  | Postpartum | Control vs. UDCA | -0,1313 | -0,5994 to 0,3368 | >0,9999 | ns |
|  |  | Control vs. PRO | -0,06412 | -0,5322 to 0,4040 | >0,9999 | ns |
|  |  | Control vs. UDCA+PRO | -0,02138 | -0,4895 to 0,4467 | >0,9999 | ns |
|  |  | UDCA vs. PRO | 0,06715 | -0,4010 to 0,5353 | >0,9999 | ns |
|  |  | UDCA vs. UDCA+PRO | 0,1099 | -0,3582 to 0,5780 | >0,9999 | ns |
|  |  | PRO vs. UDCA+PRO | 0,04274 | -0,4254 to 0,5109 | >0,9999 | ns |
|  | | | | | | |
| Total antioxidant  capacity | Prepartum | Control vs. UDCA | 0,01064 | -0,02574 to 0,04701 | >0,9999 | ns |
|  |  | Control vs. PRO | 0,003755 | -0,03262 to 0,04013 | >0,9999 | ns |
|  |  | Control vs. UDCA+PRO | 0,002901 | -0,03347 to 0,03927 | >0,9999 | ns |
|  |  | UDCA vs. PRO | -0,00688 | -0,04325 to 0,02949 | >0,9999 | ns |
|  |  | UDCA vs. UDCA+PRO | -0,007735 | -0,04411 to 0,02864 | >0,9999 | ns |
|  |  | PRO vs. UDCA+PRO | -0,0008548 | -0,03723 to 0,03552 | >0,9999 | ns |
|  | Postpartum | Control vs. UDCA | -0,01878 | -0,05515 to 0,01759 | >0,9999 | ns |
|  |  | Control vs. PRO | -0,008448 | -0,04482 to 0,02793 | >0,9999 | ns |
|  |  | Control vs. UDCA+PRO | 0,01662 | -0,01975 to 0,05299 | >0,9999 | ns |
|  |  | UDCA vs. PRO | 0,01033 | -0,02604 to 0,04671 | >0,9999 | ns |
|  |  | UDCA vs. UDCA+PRO | 0,0354 | -0,0009718 to 0,07178 | >0,9999 | ns |
|  |  | PRO vs. UDCA+PRO | 0,02507 | -0,01131 to 0,06144 | >0,9999 | ns |
|  | | | | | | |
| Immunoglobulin  G | Prepartum | Control vs. UDCA | 0,834 | -3,174 to 4,842 | >0,9999 | ns |
|  |  | Control vs. PRO | 1,37 | -2,638 to 5,378 | >0,9999 | ns |
|  |  | Control vs. UDCA+PRO | -0,938 | -4,946 to 3,070 | >0,9999 | ns |
|  |  | UDCA vs. PRO | 0,536 | -3,472 to 4,544 | >0,9999 | ns |
|  |  | UDCA vs. UDCA+PRO | -1,772 | -5,780 to 2,236 | >0,9999 | ns |
|  |  | PRO vs. UDCA+PRO | -2,308 | -6,316 to 1,700 | 0,6911 | ns |
|  | Postpartum | Control vs. UDCA | -0,644 | -4,652 to 3,364 | >0,9999 | ns |
|  |  | Control vs. PRO | 0,732 | -3,276 to 4,740 | >0,9999 | ns |
|  |  | Control vs. UDCA+PRO | -1,298 | -5,306 to 2,710 | >0,9999 | ns |
|  |  | UDCA vs. PRO | 1,376 | -2,632 to 5,384 | >0,9999 | ns |
|  |  | UDCA vs. UDCA+PRO | -0,654 | -4,662 to 3,354 | >0,9999 | ns |
|  |  | PRO vs. UDCA+PRO | -2,03 | -6,038 to 1,978 | 0,9842 | ns |
